# Beyond density: mapping the functional landscapes of macrophage-T lymphocyte niches in the tumor microenvironment

**DOI:** 10.3389/fimmu.2026.1851873

**Published:** 2026-07-02

**Authors:** Serena Zilio, Adrien Rouault Montecino, Malvina Seradj, Ilaria Marigo, Armelle Prévost-Blondel, Elisa Peranzoni, Nadège Bercovici

**Affiliations:** 1 Immunology and Molecular Oncology Diagnostics, Veneto Institute of Oncology IOV - IRCCS, Padua, Italy; 2 Université Paris Cité, CNRS, Inserm, Institut Cochin, Paris, France; 3 Department of Surgery, Oncology and Gastroenterology, University of Padua, Padova, Italy

**Keywords:** cellular neighborhood, immune cell crosstalk, spatial biology, TAM-TIL niches, tumor microenvironment (TME), tumor-associated macrophages (TAMs), tumor-infiltrating T lymphocytes (TILs)

## Abstract

The spatial arrangement of tumor-associated macrophages (TAMs) and T cells within distinct microanatomical niches is emerging as a key regulator of clinical outcome in solid tumors. Here, we propose a comprehensive review that integrates spatial insights with functional studies of macrophage and T cell heterogeneity, to elucidate how the physical crosstalk and the localization of these cells in the tumor microenvironment (TME) can impact the prognosis and the response to immunotherapy and, vice versa, how diverse immunotherapies can dynamically reshape the TME immune geography. We first review the heterogeneity of macrophages and tumor-infiltrating T lymphocytes (TILs) in TME, and the different types of TAM-TIL niches recently described by multidimensional proteomics and spatial transcriptomics, addressing their impact on tumor progression and response to immunotherapy. Building on our own work, we then dissect the anti-tumoral and pro-tumoral mechanisms operating in the different types of immune hubs. Next, we examine how tumor cues and therapeutic strategies can reprogram macrophages and T cells across functional and spatial dimensions, thereby promoting TME permissive to intraepithelial T cell infiltration and clinical response. Finally, we discuss how the integration of multi-omics and artificial intelligence are transitioning immuno-oncology from a cell-centric to a niche-centric paradigm, providing a roadmap for the design of next-generation therapies that precisely reprogram cellular dialogues within the tumor microenvironment.

## 1 Introduction

Tumor-infiltrating lymphocytes (TILs) and tumor-associated macrophages (TAMs) are two major immune populations that shape tumor evolution. While long viewed as exerting antagonistic functions, recent single-cell and spatial analyses have uncovered frequent, intimate interactions between specific TAM and TIL subsets, revealing a coordination far more dynamic than initially anticipated. These cellular dialogues can either potentiate or restrain anti-tumor immunity depending on the specific interactions and spatial organization within tumor niches, critically impacting both tumor progression and therapeutic outcomes (1, 2).

Both cell types display exceptional heterogeneity that extend well beyond classical definitions. TAMs span across a broad continuum of myeloid states, determined by ontogeny, tumor-derived signals, and metabolic cues, a complexity that the reductive M1/M2 framework fails to capture (3, 4). Similarly, TILs comprise distinct subpopulations, ranging from stem-like progenitors to terminally exhausted subsets, whose activity is heavily conditioned by their immediate surrounding environment (5).

Neither the density of TAMs nor TILs, viewed in isolation, adequately explains why some tumors progress while others regress, or why patients with comparable immune infiltrates respond so differently to the same treatment. The predictive value of the immune infiltrate lies not only in its quantity, but rather in the spatial organization and reciprocal signaling that characterize specific TAM-TIL interactions (6, 7).

The advent of high-resolution spatial technologies, including multiplex imaging and spatial transcriptomics, has revolutionized our capacity to map these interactions (8, 9). TAMs and TILs are not randomly dispersed; instead they organize into distinct microanatomical niches, such as perivascular, stromal, intraepithelial, and tertiary lymphoid structures (TLSs), each characterized by profoundly different functional and prognostic implications (10). Within these niches, a complex crosstalk of chemokines and cytokines, contact-dependent signals, and metabolic competition dictates whether the local milieu supports or suppresses anti-tumor immunity. The quantification of physical proximity and spatial organization alongside cell density seems to outperform conventional biomarkers in predicting immunotherapy response, reinforcing the idea that the cellular neighborhood, rather than just the occupant identity, governs tumor microenvironment (TME) functionality. While current infrastructures limit these technologies largely to translational research and drug development, their clinical potential is significant. As Molecular Tumor Boards become more commonly implemented in hospitals, achieving this potential will depend on encouraging closer interdisciplinary integration between clinicians and scientists to integrate complex spatial measures into routine therapeutic decisions.

This spatial perspective also reframes our understanding of therapeutic resistance. TAMs can physically trap T cells within stromal scaffolds, create immunosuppressive metabolic gradients, and drive exhaustion via prolonged inhibitory contacts in some tumor niches (11, 12). In contrast, specific TAM-TIL configurations, typical of different tumor niches, are associated with successful response to immune checkpoint blockade (ICB), suggesting that functional immune niches can underpin efficacy even in poorly infiltrated tumors (10, 13).

In this review, we integrate spatial mapping with functional studies of macrophages and T cell heterogeneity. Our goal is to describe the cellular composition and spatial architecture of “anti-tumoral” versus “pro-tumoral” niches, and to illustrate how these geographies are remodeled following a selected set of therapeutic interventions. We focus on the spatial biology of the niche as a foundational framework and propose that decoding these internal mechanisms will ultimately guide the development of more precise, next-generation therapies.

First, we outline TAM and TIL heterogeneity before examining how distinct TAM-TIL niches impact immunotherapy outcomes. Next, we dissect the reciprocal mechanisms through which these populations modulate each other’s spatial positioning and function, including the role of extracellular matrix (ECM) remodeling and treatment-induced reprogramming. Finally, we discuss how the transition from descriptive pathology to spatial multi-omics represents a fundamental shift in data dimensionality. Integrating same-section spatial metabolomics with high-plex proteomics and genomics across hundreds of thousands of cells generates a computational burden that cannot be resolved through manual observation or traditional statistical methods. Advanced modeling is, therefore, a physical necessity to quantify the complex spatial relationships that define the tumor niche. In conclusion, the advent of multi-omics, supported by increasingly sophisticated AI-driven niche identification algorithms, will drive a fundamental shift from cell-centric to niche-centric drug development, specifically engineered to reprogram cellular dialogues at the tumor-immune interface.

## 2 TAM and TIL heterogeneity

### 2.1 TIL heterogeneity

Effector CD8^+^ T cells play a central role within the TME through direct cytotoxicity and orchestration of local immune responses. Upon antigen recognition, these cells can eliminate tumor cells via perforin- and granzyme-mediated killing, and secrete key cytokines such as IFN-γ, which not only enhance tumor cell immunogenicity but also shape the surrounding microenvironment (14, 15). However, these effector functions are progressively constrained in the TME, resulting in distinct subsets of effector memory CD8^+^ T cells infiltrating solid tumors (16). In both humans and mice, a common feature is the presence of CD8^+^ T cells expressing inhibitory receptors such as PD-1, CTLA-4, TIM-3, and LAG-3, driven by chronic TCR engagement with tumor cells or antigen-presenting cells (APCs) within the TME (17, 18). These cells harbor a unique transcriptional and epigenetic signature centered on TOX, BATF and loss of T−BET/EOMES balance. Balanced T-bet/Eomes expression is important for maintaining effective anti-tumor CD8^+^ T cell responses. T-bet is primarily associated with type 1 effector programs, including cytotoxic activity, IFN-γ production, and migration into inflamed tissues, whereas Eomes shares overlapping functions with T-bet while also contributing to memory formation and long-term T cell persistence. However, excessive Eomes expression can compete with T-bet at overlapping regulatory loci, thereby weakening transcriptional programs that support effector differentiation and promoting exhaustion-associated gene expression, including TIM-3. Such an imbalance is particularly relevant in tumors, where it contributes to T cell dysfunction and impairs durable anti-tumor immunity. Effector CD8^+^ TILs also express different degrees of the transcription factor TCF1 expression, ranging from a TCF1^+^ progenitor (TpEX) subset to more terminally exhausted (TEX) populations states (19). TEX coexpresses high levels of multiple inhibitory receptors. They are characterized by reduced degranulation relative to effector cells, yet they often retain residual IFN−γ and granzyme expression but display poor long-term persistence.

Tumor specific-CD8 T cells are particularly enriched within PD-1^+^ T cells expressing CD39 (20, 21). While TEX and TpEX share some features of exhaustion, the less differentiated TpEX retains the capacity of self-renewal and expansion following PD-1 or CTLA-4 blockade, thereby enhancing anti-tumor immunity (22, 23). These TpEX exhibit memory-like features largely sustained by TCF1, which expression sustains a stem−like transcriptional program that restrains terminal differentiation and limit the upregulation of co−inhibitory receptors (24). Indeed, enforced TCF1 expression biases TEX toward this progenitor state with improved polyfunctionality, while preserving the capacity to enter the exhaustion trajectory. Importantly, higher densities of TCF1^+^ PD-1^+^ CD8^+^ T cells in patients correlate with durable response to ICB (5, 25).

Tissue-resident memory T (TRM) cells constitute another TILs subset that has emerged as critical for tumor control (26, 27). These cells express retention receptors such as CD103, an integrin that binds epithelial E-cadherin to promote retention within epithelial layers, as well as CD69 or the integrin CD49a. TRMs contribute to tissue immunosurveillance through their ability to rapidly mediate cytotoxicity upon activation while amplifying the initial immune response by recruiting other immune cells (28). These CD103^+^CD8^+^ TRMs are enriched in highly differentiated tumor-reactive CD8^+^ T cells (20) and their high density has been correlated with improved survival and response to immunotherapy across multiple cancer types including head and neck, breast and non-small cell lung (NSCLC) cancers.

Besides effector CD8^+^ T cells, multiple CD4^+^ T cell subsets infiltrate tumors, where they can exert divergent and sometimes opposing functions. Regulatory CD4^+^ T cells (Tregs) are typically enriched in tumors and represent a major immunosuppressive population (29). Within the TME, Tregs upregulate inhibitory receptors such as CTLA-4, PD-1, LAG-3 and TIGIT and metabolically adapt to survive under conditions of hypoxia and nutrient deprivation. They favor tumor growth and suppress anti-tumor T cell responses through several mechanisms. Notably, Treg depletion has been shown, by our group and others, to promote anti-tumor polarization of monocytes and macrophages (30–32). Clinically, a high Treg density or a low ratio of CD8^+^ T cells to Tregs generally correlates with poor prognosis and reduced response to immunotherapy (33). Nevertheless, by limiting chronic inflammation, Tregs may also indirectly restrain tumor progression underlying the complexity of Tregs functions at different tumor stages (34, 35).

In parallel, conventional CD4^+^ T cells have received increasing attention in recent years. Despite their critical role in effective anti-tumor immunity, their functional impairments remain less characterized than those of CD8^+^ T cells (36). CD4^+^ T cells can differentiate into Th1 cells, which, in addition to enhancing CD8^+^ T cell cytotoxicity, promote macrophage polarization toward inflammatory states and inhibit tumor angiogenesis. In contrast, the role of Th2-cells may be context-dependent. IL-4 produced by Th2 cells has been linked to pro-tumoral effects, notably through the polarization of macrophages. For example, in breast cancer models, Th2-derived IL-4 directly promotes pro-tumorigenic TAM functions by inducing factors such as Arginase-1 (Arg-1) and TGF-β, as well as pro-metastatic mediators including EGF (31). In addition, in a glioma model IL-4 triggers IGF-1 production in macrophages, thereby activating the PI3K pathway in tumor cells (37). Nevertheless, in the absence of TGF-β signaling, Th2 cells have been shown to promote tumor vasculature remodeling, leading to hypoxia-associated cancer cell death (38). Another subset, Th17 cells, characterized by RORγt expression and IL-17 and IL-22 production, display marked functional plasticity, exerting both pro- and anti-tumoral activities depending on the tumor context (39). Th17 cells may support tumor progression by promoting myeloid cell recruitment and survival, as well as tumor cell growth. Conversely, they can also contribute to anti-tumor immunity by facilitating the emergence of IFN-γ-producing Th1-like cells and by recruiting dendritic cells (DCs) to the tumor site. Finally, Bcl-6^+^, CXCR5^+^, IL-21^+^ T follicular helper (Tfh) cells support anti-tumor immunity by promoting the formation of TLSs (40, 41). The presence of Tfh cells and TLSs in several non-lymphoid tumors, including colorectal, lung, and breast cancers, has been associated with improved patient survival (42, 43). Tfh cells also express high levels of PD-1 and have recently been shown to promote the priming of anti-tumor CD8^+^ T cells in tumor-draining lymph nodes through IL-4 secretion after anti-PD-1 treatment (44). Altogether, these findings indicate that the composition of CD4^+^ T cell subsets can strongly influence anti-tumor immunity and the efficacy of immunotherapies.

#### 2.1.1 Spatial heterogeneity of T cells

From the introduction of the Immunoscore concept two decades ago (7), the clinical impact of the spatial localization of T cells within the TME, in addition to their density (45), has been increasingly recognized. Three main T cell distribution patterns are usually described in human cancers: *immune inflamed*, *immune excluded* and *immune desert*. Immune inflamed tumors display high numbers of T cells, distributed both in the stroma and inside tumor nests in close proximity to tumor cells, and are associated with favorable prognosis and high sensitivity to ICB. Non-inflamed tumors fall in two categories: immune excluded, where, regardless of their abundance, T cells are confined to the stroma surrounding tumor nests, either at its margins or across the whole tumor area (46), and immune desert, which show a very low density of TILs across all compartments. In both cases, patients face poor prognosis and show resistance to immunotherapy, unless T cell infiltration and migration are increased by other therapeutic interventions (47–51). In addition, T cells can also be found inside TLSs (52), which are generally related to good clinical outcomes.

The study of the spatial distribution of TIL subsets is still in its early stages (53). Both TEX and TpEX CD8^+^ T cell subsets are significantly more prevalent in stromal areas than intra-tumoral regions, with terminally exhausted cells often outnumbering progenitors. In fact, TCF1^+^ progenitors are preferentially localized in specific niches, particularly within the stromal barrier, in regions enriched in CD11b^+^CD11c^+^MHC-II^+^ APCs that are likely crucial for their effector differentiation (54, 55). CXCL16, and IL-15 expressed by CCR7^+^ DCs near perivascular regions in the stroma, have been identified as key factors driving the positioning and survival of recently activated CXCR6^+^ TCF1^-^ effector cells (56).

Multiplex imaging revealed that Tregs accumulate in stromal regions and at the tumor-stroma boundary in different carcinomas, where chemokine gradients and cancer-associated fibroblast (CAF) networks facilitate their retention (57, 58). Interestingly, high islet−infiltrating Treg densities correlate with immunosuppression and poor outcome, whereas a predominance of stromal CD4^+^ T cells (including Th1 cells) has been linked to a more favorable prognosis in tumors such as NSCLC (59, 60). By contrast, CXCL13^+^ CD4^+^ Tfh−like cells are frequently concentrated in or around TLSs in the peritumoral stroma and at tumor margins. Together, the balance between suppressive/Treg−rich, stromal−locked versus effector/Tfh−rich, TLS−forming architectures tightly regulates clinical outcome and response to therapy (59). In addition, beyond shaping interactions with TAMs, the heterogeneous spatial distribution of TILs within the TME may also critically influence interactions between distinct TIL subsets themselves. For instance, the accumulation of Tregs within tumor nests may locally suppress cytotoxic CD8^+^ T cell activity, thereby impairing anti-tumor immunity, whereas their presence in stromal regions can limit chronic inflammation driven by activated T cell subsets such as Th17 cells. In parallel, TRM cells may contribute to the recruitment and spatial organization of additional immune cells, including other T cells, thereby participating in the establishment of local immune niches within the TME.

### 2.2 TAM heterogeneity

TAMs represent the predominant immune cell population in most solid tumors, and their abundance is usually correlated with poor prognosis (3, 45). However, TAMs are phenotypically and functionally diverse and can also exert anti-tumor activities. Harnessing their protective mechanisms and reprogramming them therefore represent a promising therapeutic strategy (61).

#### 2.2.1 Ontogenic diversity

A large proportion of TAMs in murine and human tumors originate from circulating monocytes and are recruited during tumor development. The TAM pool also includes resident-tissue macrophages (RTMs) that populate the tissue before tumor initiation, and persist in the TME during progression. RTMs originate either from embryonic precursors maintained by local self-renewal or from circulating monocytes recruited before tumor development. Embryonic macrophages are generated in three successive waves from erythromyeloid progenitors, originating first within the yolk sac and hematogenic endothelium, and subsequently within the aorta-gonad-mesonephros region and fetal liver (4). Macrophages derived from postnatal hematopoiesis originate primarily in the bone marrow (BM). Here, hematopoietic stem cells differentiate into common-myeloid progenitors, giving rise to monocytes via two parallel differentiation pathways: the granulocyte-monocyte progenitors, which also generate polymorphonuclear leukocytes, and the macrophage-dendritic cell progenitors, that also produce conventional and plasmacytoid DCs (62).

The relative contribution of fetal-derived versus adult hematopoiesis-derived macrophages to the RTM pool depends on both niche accessibility and organ type. Closed niches, such as microglia in the brain or alveolar macrophages in the lungs, only admit fetal-derived macrophages (63, 64), while open niches, such as the intestine or lung interstitium, allow the progressive postnatal replacement of embryonic cells by monocyte-derived macrophages at an organ-dependent rate (65). Importantly, residency within physiological tissue niches, before tumor onset, shapes monocyte-derived macrophages to phenotypically match their embryonic counterparts (66, 67). RTMs actively contribute to carcinogenesis displaying distinct phenotypic, transcriptional, and even functional signature compared to monocyte-derived TAMs recruited during tumor growth (68–72). Moreover, due to their ability to be maintained independently of BM-derived monocytes, it is not surprising that RTMs have been found to express a self-renewal gene signature in metastatic ovarian cancer (69).

Beyond their unique origins, these two lineages may also segregate into distinct spatial niches, largely dictated by how monocyte-derived TAMs are recruited to the TME. In particular, the CCL2/CCR2 axis governs the recruitment of peripheral monocytes into tissues, maintaining homeostatic equilibrium at baseline and driving rapid influx during inflammation. Mechanistically, a systemic CCL2 gradient triggers the egress of classical CCR2^+^ monocyte from the BM, while CCR2 signaling activates surface integrins to mediate endothelial adhesion and extravasation. This pathway is subverted in tumors, where a chronically inflamed TME induces massive CCL2 production by tumor cells, CAFs, mesenchymal stem cells, and CCR2^+^ TAMs themselves (73, 74). This drives a massive influx of monocytes that differentiate into TAMs in response to local host- and tumor-derived signals. Accordingly, disrupting the CCR2-CCL2 signaling via genetic targeting or neutralizing antibodies largely abrogates monocyte recruitment and subsequent TAM differentiation (70, 75).

#### 2.2.2 Phenotypic and functional heterogeneity of TAMs - beyond M1/M2 classification

Historically, macrophage activation has been simplified into a binary dichotomy that mirrors the Th1/Th2 nomenclature, representing opposite extremes of a functional spectrum. M1 (classical) activation is triggered by IFN-γ and toll-like receptor (TLR) agonists, such as LPS, and results in a transcriptional profile dominated by pro-inflammatory mediators, including STAT1, NF-kB, IL-1β, IL-12, and TNF-α, alongside the induction of inducible nitric oxide synthase (iNOS). This phenotype is generally associated with intracellular pathogen killing and tumor cell destruction. M2 (alternative) activation is primarily induced by IL-4 and IL-13, with a transcriptional program characterized by the expression of STAT6, PPARγ, Arg-1, and CD206, leading to high production of IL-10 and TGF-β while suppressing pro-inflammatory signaling. This activation state is associated with wound healing and immunosuppression. While this classification provides a useful prognostic shorthand, large-scale transcriptomic analyses of TAMs suggest that these extremes fail to capture the true complexity of the TME. Current research has transitioned toward a spectrum or continuum model of macrophage activation, where TAMs may display hybrid transcriptional programs, in some cases co-expressing both M1 and M2 markers, as discussed below (76–79).

It is now recognized that distinct human TAM subsets correlate with highly divergent clinical outcomes and immunotherapy responses. Broadly, TAMs expressing markers such as CD163, CD206, CD204, SPP1 (osteopontin), MARCO, TREM2, and STAB1 (stabilin-1), usually associated with an M2-like phenotype, or less characterized antigens like GPNMB, Siglec-10, FABP5, INHBA, VCAN, and DC-SIGN, are correlated with poor prognosis and metastasis in multiple solid tumors. Conversely, CXCL9^+^ and CXCL10^+^ TAMs, usually associated with an M1-like phenotype, alongside multinucleated giant cells (MGCs) expressing Chitinase-1 (CHIT1), generally track with favorable patient outcomes (61, 80, 81).

However, assigning a specific phenotype to a definitive clinical outcome remains highly complex, as identical phenotypes can behave very differently depending on the tumor type or spatial localization.

For instance, Ray and colleagues identified a protective TAM population expressing CD206, a marker commonly associated with the M2-like type, yet actively participating in the recruitment of anti-tumor immune cells (CD8^+^ T cells, NK cells, and cDC1) (82). Similarly, FOLR2^+^ TAMs exhibit striking disease-specific duality: in hepatocellular carcinoma, they co-localize with Tregs to sustain an immunosuppressive niche, while in breast cancer, they can actively stimulate effector CD8^+^ T cells and correlate with improved patient survival (72, 83, 84). Likewise, although TREM2^+^ TAMs are described as immunosuppressive and pro-tumoral in most solid tumors, they were found to correlate with a good prognosis in head and neck carcinoma (85, 86). Similarly, while the enzyme IL-4 induced gene 1 (IL4I1) is widely expressed by TAMs and associated with immunosuppressive functions and poor clinical outcomes, IL4I1^+^ TAMs have also been observed in close proximity to tumor cells, actively phagocytizing dying cells in colorectal cancer (CRC) and correlating with anti-PD-1 response in breast cancer (83, 87, 88).

Spatial architecture can also heavily influence cell function. Subcapsular sinus CD169^+^ macrophages correlate with favorable outcomes when restricted to tumor-draining lymph nodes in melanoma, endometrial, bladder, esophageal, and breast cancers (89–93). Yet, when these same cells infiltrate tertiary lymphoid-like structures (TLLSs) within primary breast tumors, they drive worse clinical outcomes by driving the maturation of the TLSs toward an immunomodulatory state and a Foxp3^+^ T cell signature (90).

Over the past decade, the same TAM subsets associated with poor survival, characterized by the expression of TREM2, SPP1, CD163, or CD206, have consistently emerged as correlates with resistance to immune checkpoint inhibitors. In contrast, baseline macrophage signatures enriched in PD-1 ligands (PD-L1, PD-L2), pro-inflammatory cytokines and chemokines or interferon-stimulated genes tend to characterize tumors of patients who have better prognosis and also respond to ICB (61, 81, 94).

The integration of multi-cancer datasets and the advancement of high-resolution RNA sequencing have made it possible to categorize distinct TAM subsets according to their conserved functional and transcriptional programs. Examples include: *IFN-TAMs*, enriched in IFN-regulated genes (*CXCL10* and *ISG15* but also immunosuppressive molecules like *IDO1, IL4I1*, and *CD274*) and costimulatory markers (*CD40*, CD86, and MHC-II); *Inflam-TAMs*, enriched in inflammatory cytokines including *IL1B*, *CXCL1/2/3/8*, *CCL3*, and *CCL3L1*; *Angio-TAMs* that interact with fibroblasts and display a pro-angiogenic signature including *VEGFA*, *SPP1, VCAN*, *FCN1* and *THBS1*; *Lipid-associated TAMs (LA-TAMs)*, characterized by the expression of lipid-related genes (*APOC1, APOE, ACP5*, and *FABP5*) and a distinctive enrichment in lipid metabolism and oxidative phosphorylation pathways; *regulatory TAMs (Reg-TAMs)*, showing high expression of *ARG1*, *MRC1*, and *CX3CR1*; *resident-tissue macrophages (RTM-TAMs)*, that resemble normal resident macrophages and express *LYVE1*, *HES1*, and *FOLR2; Prolif-TAMs*, characterized by the expression of proliferation marker (*MKI67*) and cell cycle genes such as *CDK1* and *CDC45* (95).

The advent of high-resolution analytical techniques (CyTOF, scRNA-seq, multiplex imaging) has further confirmed the complexity and heterogeneity of TAMs, revealing variability in TAM subpopulations among individuals, cancer types, disease stages, pre- or post-treatment conditions, and even within the same tumor, thereby rendering the M1/M2 classification obsolete.

#### 2.2.3 Spatial heterogeneity of TAMs

The phenotype of TAMs is not the only determinant of the prognostic or predictive impact of these cells in human cancer. As a matter of fact, the spatial localization of TAMs within the TME and their interactions with other cell types are crucial for their influence on clinical outcomes and therapeutic efficacy (81, 96, 97). In hypoxic areas, TAMs are generally associated with immunosuppression, angiogenesis, and poor clinical outcomes (80, 98, 99). Likewise, perivascular macrophages located in close proximity to blood vessels often characterized by TIE-2 and LYVE-1 expression, are specialized in supporting angiogenesis and tumor cell dissemination and are consequently associated with poor prognosis (83, 100, 101).

RTMs and monocyte-derived TAMs can also occupy distinct regions within the tumor. For instance, in breast cancer, Nalio Ramos and colleagues identified FOLR2^+^ resident macrophages and TREM2^+^ monocyte-derived macrophages in the stroma and inside tumor nests, respectively (72). The spatial distribution of RTMs can also change during tumor progression: in lung cancer, for instance, they initially accumulate in close proximity to malignant cells at early stages of tumor formation, before redistributing toward the periphery of the TME (102).

However, establishing a universal “spatial rule” remains elusive. The prognostic value of TAM localization in the peritumoral stroma, inside tumor nests or within tertiary lymphoid structures is highly heterogeneous across different malignancies, suggesting that the clinical impact is dictated by the specific biological context of each tumor type. For instance, while the TIM4^+^FOLR2^+^ TAM subset in TLSs is associated with T cell infiltration and better prognosis (103), the CD169^+^ population in these same structures has a negative prognostic value (90). In a similar way, TAMs expressing CD163 or CD206 in stromal regions are generally linked to poor survival, but a stromal FOLR2^+^ subset is correlated with positive outcomes in breast cancer (72, 83).

Unlike T cells, the presence of TAMs inside tumor nests is not necessarily beneficial. Indeed, the presence of CD68^+^COX2^+^ or CD163^+^ TAMs in tumor nests of breast cancer is associated with poor prognosis (104, 105).

Collectively, these observations underline that the clinical significance of TAMs is defined by the combination of their phenotype and their spatial localization within the TME, and the two must be interpreted in conjunction.

### 2.3 ECM-mediated regulation of TAM and TIL localization and function

In many solid malignancies, aberrant ECM deposition by CAFs and other stromal cells increases matrix density, stiffness, and fiber alignment, thereby shaping both the localization and function of immune cells within the TME. These structural alterations generate physical barriers that impede effective lymphocyte infiltration into the tumor parenchyma, contributing to the characteristic “immune-excluded” phenotype (47, 106–108).

Mechanistically, dense and cross-linked collagen networks, including collagen VI-rich matrices, restrict T cell motility and access to tumor regions (106, 109, 110). Beyond acting as passive barriers, ECM components actively regulate immune cell behavior through biophysical and biochemical interactions. ECM molecules engage receptors expressed by lymphocytes, including CD44, integrins, and leukocyte-associated immunoglobulin-like receptor 1 (LAIR-1), thereby modulating adhesion dynamics, cytoskeletal remodeling, mechano-transduction pathways, chemotaxis, and retention within specific niches of the TME (110). High collagen density can directly suppress TCR signaling through inhibitory receptors such as LAIR-1, reducing effector functions and promoting T cell exhaustion (111).

CAFs initiate this reciprocal ECM-immune loop by orchestrating excessive ECM deposition through the production of collagens and fibronectin. In gastric cancer peritoneal metastases, ECM-driven immunosuppression associated with hyaluronic acid (HA) accumulation limits the efficacy of anti-PD-1 therapy. In fact, HA engages CD44 on T cells, promoting Treg differentiation and dampening cytotoxic responses. Importantly, disrupting this HA-CD44 signaling axis reduces immunosuppression and restores effector T cell activity, thereby enhancing responsiveness to immunotherapy (112).

In parallel, the ECM selectively supports the accumulation and stability of Tregs by providing chemokine reservoirs and adhesion platforms favoring their retention within the tumor stroma. ECM-TIL interactions may also influence the formation and maintenance of TLSs, which can be disrupted in matrix-rich tumors due to excessive stiffness and altered biochemical gradients (113). Proteoglycans such as versican and hyaluronan, frequently enriched in TAM-rich regions, further contribute to immunosuppression by shaping cytokine gradients and dampening effector T cell responses (114).

TAMs themselves actively remodel the ECM through the secretion of matrix metalloproteinases (MMPs), cathepsins, and lysyl oxidases, as well as through the deposition of fibrillar collagens, fibronectin, and proteoglycans. By modifying collagen architecture and matrix topography, TAMs can generate tumor regions that are less permissive to T cell recruitment and migration (115). Conversely, ECM-derived biochemical and biomechanical cues feed back onto TAMs and reinforce their polarization toward pro-tumoral and immunosuppressive phenotypes. Increased matrix stiffness and altered integrin signaling activate mechano-transduction pathways involving focal adhesion kinase (FAK), YAP/TAZ, and TGF-β signaling, which sustain TAM survival, promote anti-inflammatory transcriptional programs, and enhance the secretion of immunosuppressive mediators supporting angiogenesis, fibrosis, immune evasion, and metastatic dissemination (116, 117). Consistent with this concept, Puttock and colleagues demonstrated that macrophages cultured on decellularized metastatic ECM scaffolds derived from ovarian cancer metastases acquire transcriptional programs resembling human TAMs, providing mechanistic evidence that ECM components alone can educate macrophages toward tumor-supportive states (118).

Single-cell RNA sequencing of primary hormone receptor-positive breast cancers revealed an enrichment of SPP1^+^ TAMs within tumors displaying high TIL infiltration. These macrophages exhibit a strong ECM-remodeling signature characterized by SPP1 and fibronectin expression, together with activation of matrix-organizing pathways. Spatial transcriptomic analyses further showed that these TAMs localize within ECM-rich niches in close proximity to TILs, where they engage T cells through SPP1-mediated adhesion and signaling pathways. These findings suggest that macrophage-driven matrix remodeling shapes both the physical and biochemical properties of the TME, thereby influencing immune cell localization, function, tumor progression, and therapeutic responses (119).

Together, these observations support the existence of an ECM-TAM-TIL axis in which matrix remodeling suppresses cytotoxic T cell function, favors regulatory immune populations, and further entrenches macrophage-driven immunosuppression. This ECM-TAM-lymphocyte axis represents a key determinant of tumor immune exclusion and resistance to immunotherapy, particularly to ICB.

## 3 TAM-TIL interactions in treatment-naive tumors

While individual immune cell counts provide a foundational overview of the tumor-immune microenvironment, they frequently fail to capture the complex dialogue between immunosuppressive and effector cells that ultimately dictates clinical outcomes. Understanding the spatial proximity and reciprocal interactions within TAM-TIL niches offers a far more robust predictor of prognosis and response to ICB than the density of either cell type alone. The importance of viewing these localized immune niches as the primary determinants of therapeutic success or failure is exemplified by the Immunoscore-IC, that quantifies not only the spatial density but also the proximity of both cytotoxic CD8^+^ T cells and PD-L1^+^ cells (primarily macrophages and tumor cells) and provides a superior predictive indicator of immunotherapy response compared to traditional biomarkers (120, 121).

### 3.1 TAM-TIL niches: phenotypic diversity, spatial organization and clinical significance

**Figure 1:**
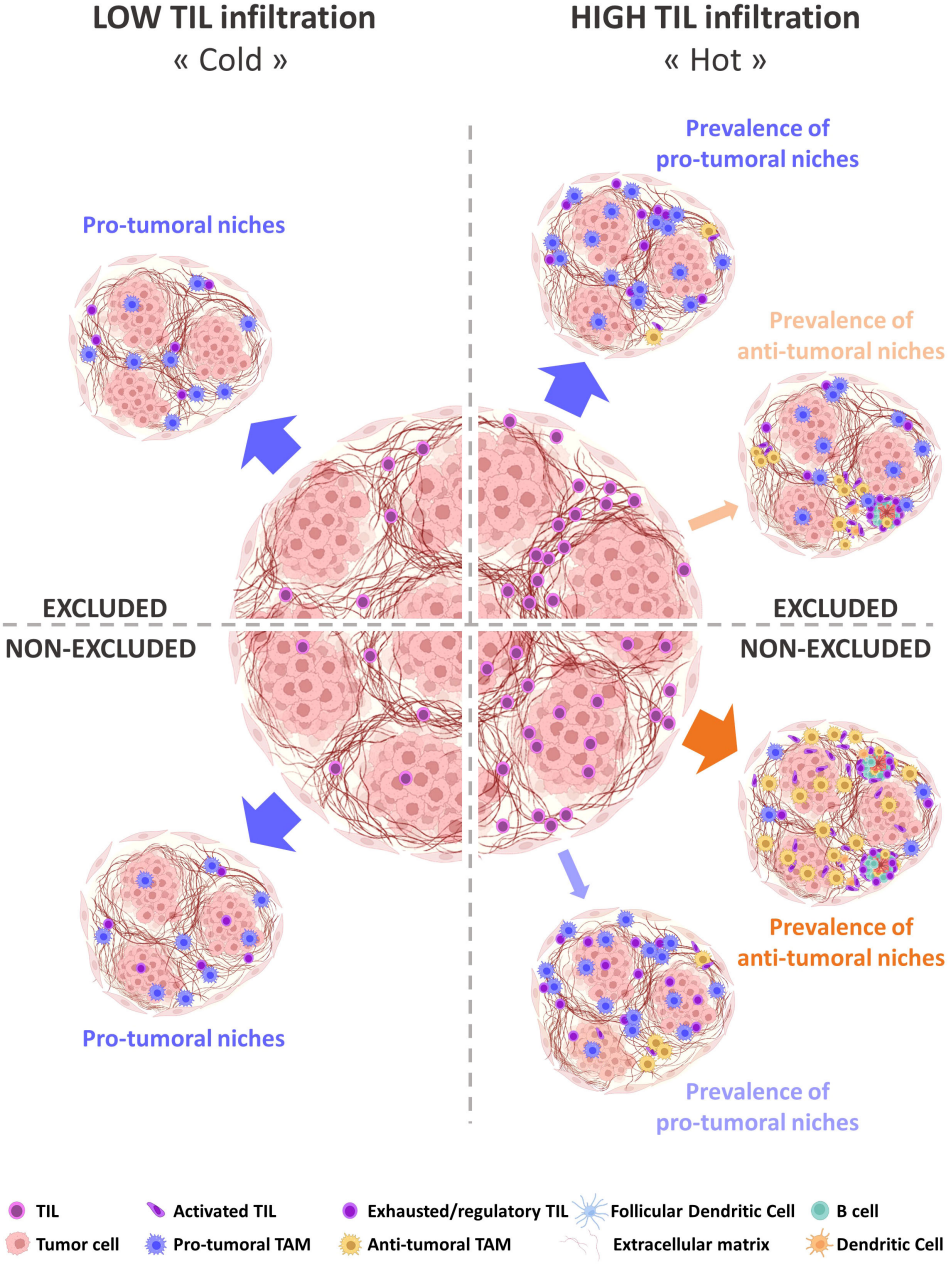
Cold, hot and immune exclusion: from a T cell-centric to a niche-focused view. The current view of “cold” and “hot” tumors mainly relies on the abundance of TILs in the TME. Cold (desert) tumors are typically characterized by a low level of TILs, whereas hot (inflamed) tumors contain a higher TIL density. The spatial distribution of TILs further determines whether a tumor is classified as immune “excluded”, with lymphocytes confined to the surrounding stroma, or “non-excluded”, with TILs infiltrating tumor islets. We propose to expand this classification by taking into account the type and abundance of TAM-TIL niches, including those inside TLSs, present in the TME in addition to the spatial TIL distribution. The presence of anti-tumoral TAM-TIL niches (yellow anti-tumoral TAMs/purple migrating activated TILs) is more likely in hot, non-excluded tumors (higher probability shown by the thicker orange arrow) than in hot, excluded ones (lower probability shown by the thinner light orange arrow), and its prevalence is generally associated with a better clinical outcome. On the other hand, the presence of pro-tumoral TAM-TIL niches (blue pro-tumoral TAMs/purple round exhausted/regulatory TILs) is almost exclusive in cold tumors and more likely in hot, excluded tumors (thicker blue arrow) than in hot, non-excluded ones (thinner light blue arrow), its prevalence being generally associated with a worse prognosis. Created with BioRender.com.

Recent technological breakthroughs in spatial transcriptomics and multiplexed proteomics are unraveling the reciprocal interactions and spatiotemporal coordination between different tumor-infiltrating cell types and their impact on anti-tumor immunity. In this context, several TAM-TIL niches with distinct impact on clinical outcome have been described in treatment-naïve tumors. While the current classification of tumors into immune desert, immune excluded, and immune inflamed phenotypes provide a useful topographical framework based on TIL density, it fails to account for the functional diversity within these categories. In **Figure 1**, we propose a paradigm shift from a TIL-centric to a niche-centric perspective. This view integrates the spatial proximity and activation states of TAMs and TILs, illustrating how specific “TAM-TIL hubs” can tip the balance toward either anti-tumoral or pro-tumoral outcomes. The different types of TAM-TIL hubs are also summarized in **Table 1**.

**Table 1:** Different types of TAM-TIL hubs.

Type of TAM-TIL hub	TAM subsets	TIL subsets	Other cells	Localization of TAM/TIL hub within the TME	Impact on patient prognosis or response to ICB	Tumor type	Ref
Immunosuppressive TAM-TIL hub	SPP1^+^ TAMs	CD44^+^ T cells		tumor-stroma interface	associated with immunosuppression and immune exclusion	NSCLC	(122)
Immunosuppressive TAM-TIL hub	NECTIN2^+^ TAMs	TIGIT^+^ T cells		tumor-stroma interface	associated with immunosuppression and immune exclusion	NSCLC	(122)
Immunosuppressive TAM-TIL hub	HLA-E^+^ TAMs	CD8^+^ T cells		tumor-stroma interface	associated with immunosuppression and immune exclusion	NSCLC	(122)
Immunosuppressive TAM-TIL hub	SPP1^+^ TAMs	CD8^+^ Tex cells expressing PD-1, TIGIT, CTLA-4, LAG-3, and CXCL13		not specified	linked to the induction of immunosuppression	mGC	(123)
Immunosuppressive TAM-TIL hub	TREM2^+^SPP1^+^ TAMs	CD8^+^ Tex or Tregs expressing CD44 or ITGA4/ITGB1		not specified		ESCC	(124)
Immunosuppressive TAM-TIL hub	TREM2^+^SPP1^+^ TAMs expressing CD166 and GALECTIN-9	CD8^+^ Tex cells expressing CD44, TIM-3 and CD6		not specified	associated with poor prognosis	HNSCC lymph node metastases	(125)
Immunosuppressive TAM-TIL hub	SPP1^+^ TAMs enriched for coagulation and cholesterol homeostasis pathways	CD8^+^ T cells expressing CD44	MMP11^+^ CAFs and tumor cells expressing *TGFBI* and *PERP*	tumor periphery (within 50 µm of the tumor boundary)	linked to the induction of immunosuppression	CRC	(126)
Immunosuppressive TAM-TIL hub	SELENOP^+^ TAMs enriched for inflammatory responses and TNF signaling	T cells via the B2M–KLRD1 axis	tumor cells and transitional goblet cells expressing *REG1A* and *LCN2*	tumor periphery (within 50 µm of the tumor boundary)	linked to the induction of immunosuppression	CRC	(126)
Immunosuppressive TAM-TIL hub	CD206^+^CD11c^+^ TAMs	CD8^+^ T cells		stromal regions	linked to reduced CD8+ T cell migration and intraepithelial infiltration	NSCLC and BC mouse models	(12)
Hypoxic hubs	CD206^+^ARG1^+^ TAMs	PD-1^+^TIM-3^+^CD8^+^ Tex cells	tumor cells	hypoxic tumor areas	positively correlated with higher glioma grade and aggressive GBM subtype, which is strongly linked to immunosuppression and poor patient outcome	GBM	(99)
Hypoxic hubs	MS4A7^+^APOE^+^ TAMs	CD8^+^ Tex cells		hypoxic tumor areas	associated with protection rather than elimination of the tumor	melanoma and BC mouse models	(98)
TAM-Treg hub	CD169^+^ TAMs	Tregs	Bregs	TLS-like structures	independent negative prognostic factor	BC	(90)
TAM-Treg hub	Monocytes and TAMs	Tregs and TH17 cells	Neutrophils, tumor cells and CAFs	interface between the tumor and the luminal margin, especially in highly necrotic regions	immunosuppressive and pro-tumorigenic hub	CRC	(127)
TAM-Treg hub	CD163^+^ TAMs	Tregs and CD8^+^ T cells		not specified	associated with the poorest survival and to treatment failure	ccRCC	(128)
TAM-Treg hub	CD163^-^ and CD163^+^ TAMs	Tregs	B cells		associated with worse patient survival	NSCLC adenocarcinoma	(130)
TAM-Treg hub	M2 TAMs	Tregs and CD8^+^ T cells	PMN-MDSCs, vessel cells and CD90^+^ cells	stromal regions	highly immunosuppressive phenotype, correlated with worse prognoses in other cancers	HNSCC	(131)
Intraepithelial immunosuppressive TAM-TIL hub	CD68^+^CD163^+^ TAMs	T cells including Tregs	neutrophils and tumor cells	within tumor islets	associated with immunosuppression	NSCLC	(132)

Inflammatory chemokine-driven TAM-TIL hub	CXCL10^+^ macrophages expressing C1QA, C1QB, LYZ, CD14, CD68, CD163, S100A9, CCL18, CSF1R and TLR22	TCF7^−^CD8^+^ T cells expressing CCL5, CD8A, CD2 and LAG-3	TCF7^+^PD-1^+^CD8^+^ T cells, activated CCR7^+^LAMP3^+^ dendritic cells, CCL19^+^ fibroblasts	stromal regions, often at the peritumoral edge	strongly associated with favorable PD-1-blockade outcome	NSCLC	(133)
Inflammatory chemokine-driven TAM-TIL hub	CXCL10^+^/CXCL11^+^ TAMs	IFNγ^+^ and CXCL13^+^ T cells	tumor cells	not specified	enrichment of this hub in dMMR CRC tumors, known to be immunogenic and responsive to immunotherapy	CRC	(127)
Inflammatory chemokine-driven TAM-TIL hub	Pro-Inflam TNFSF10^+^ TAMs expressing HLA-C, CXCL9, CXCL11, TNFSF10 and PD-L1	Tex expressing LILRA1/B1/B2, CXCR3, TNFRSF10A and PD-1		not specified	significantly associated with response (complete response/partial response + long-term survival with stable disease) to ICI	BC, CRC, PDAC, ESCC/ESCA, STAD, THCA, RC, HCC, NSCLC	(134)
Inflammatory chemokine-driven TAM-TIL hub	CXCL9/CXCL10/CXCL11+TAB1+ TAMs	Clonally expanded CD8+ T cells expressing PRF1, GZMA, GZMK	B cells and endothelial cells	close to tumor cells and within gut-associated lymphoid tissues	Potential anti-tumor function	CRC	(126)
Inflammatory chemokine-driven TAM-TIL hub	CCR2^+^ Mono-like Macs, IL1β^+^CCL3/4^+^PD-L1^+^ Inflam Macs, and CXCL1^+^CXCL3^+^CCL2^+^ lipid-associated macrophages (LAM2s)	PD-1^+^ CD4^+^ TH1, PD-1^+^CD8^+^ TEX, Tregs	neutrophils, mRegDCs, AXL DCs, lymphatic ECs	not specified	positively correlated with response to ICIs	BC, HNSCC	(135)
C1QC^+^ TAM-TIL hub	C1QC^+^ RTMs have antigen-presenting capacity, a low M2 score and a low innate anti-PD-1 resistance signature (IPRES)	CD4^+^ T cells expressing CD38, CD57, GZMB, TNFα and PD-1	CAFs, Tregs, CD8+ T cells	not specified	more abundant in CRC responders to anti-PD-1 regardless of microsatellite status - pts with shorter median C1QC^+^ RTM-CD4^+^ T cell distances display a greater survival benefit than those with longer median distances	CRC	(136)
FOLR2^+^ TAM-TIL hub	FOLR2^+^ TAMs	CD8^+^ T cells expressing *GZMA, GZMB, GZMK, PFR1, KLRB1* and *KLRD1*	CD31^+^ endothelial cells	mainly located in the stroma, especially in perivascular regions	associated with a better outcome BC	BC	(72)
FOLR2^+^ TAM-TIL hub	FOLR2^+^ RTMs	CD4^+^ T cells, CD8^+^ T cells, Tregs	CD38^+^CD138^+^ plasma cells, DCs, FAP^+^ fibroblasts	peritumoral stroma as well as benign tissue	not specified	CRC	(83)
FOLR2^+^ TAM-TIL hub, TLS hub	FOLR2^+^TIM-4^+^ TAMs	CD3^+^ T cells	B cells, CD34^+^ endothelial cells	T cell zones of TLS	associated with better prognosis, TIL infiltration and MSI status	BLCA, BC, CRC, HNSCC, NSCLC, OV, SKCM, UCEC	(103)
TAM-TIL TLS hub, Inflammatory chemokine-driven TAM-TIL hub	CXCL10^+^STAT1^+^ IFN-macrophages expressing PD-L1	CD4^+^ TFH cells expressing PD-1	B cells (plasmablasts, BREG, and GC B cells) and mQuiescDCs	TLS	positively correlated with response to ICIs in BC and HNSCC	HNSCC	(135)
TAM-TIL TLS hub	CD163^−^ macrophages	CD8^+^ T cells (including Granzyme B^+^ T cells)	B cells	TLS	associated with better survival	ccRCC	(128)
TAM-TIL TLS hub	CD163^+^ TAMs	Tregs	CD4^+^ T cells, CD8^+^ T cells, CD4^+^PD-1^+^ Tfh cells, B cells, CD56^+^ NK cells	intratumoral and peritumoral TLS	patients having more intratumoral than peritumoral TLS experience a better clinical outcome	CRC liver metastases	(129)
TAM-TIL TLS hub	monocytes and M1 TAMs	CD4^+^Foxp3^-^ and CD8^+^ T cells	Plasma cells, CD90^+^ MSC-like cells, vessel cells	stromal TLS	strongly associated with a good prognosis in HNSCC	HNSCC	(131)
Intraepithelial PD-L1^+^ TAM-TIL hub	PD-L1^+^CD11c^+^ TAMs	PD-1^+^CD8^+^ TILs	PD-L1^+^CD11c^+^ DC	within tumor islets	associated with improved survival in HGSOC	HGSOC	(137)
Intraepithelial PD-L1^+^ TAM-TIL hub	CD68^+^CD206-PD-L1^+^ TAMs	CD8^+^ TILs		within tumor islets	associated with the presence of CD8+ TILs and good patient outcome in BC	BC	(48)

BC, breast cancer; BLCA, bladder urothelial carcinoma; ccRCC, clear-cell renal cell carcinoma; CRC, colorectal cancer; ESCC/ESCA, esophageal squamous cell carcinoma/esophageal adenocarcinoma; GBM, glioblastoma; HCC, hepatocellular carcinoma; HNSCC, head and neck squamous cell carcinoma; HGSOC, High-Grade Serous Ovarian Cancer; mGC, metastatic gastric cancer; NSCLC, non-small cell lung cancer; OV, ovarian cancer; PDAC, pancreatic adenocarcinoma; RC, renal carcinoma; SKCM, skin cutaneous melanoma; STAD, stomach adenocarcinoma; THCA, thyroid carcinoma; UCEC, uterine corpus endometrial carcinoma.

#### 3.1.1 TAM-TIL niches associated with poor prognosis/resistance to ICB

##### 3.1.1.1 Immunosuppressive TAM-TIL hubs

A recent study identified three different TAM-TIL axes, enriched at the tumor-stroma interface, that drive immunosuppression and immune exclusion in NSCLC. One involves SPP1^+^ TAMs that interact with CD44^+^ T cells inducing exhaustion, another one includes NECTIN2^+^ TAMs that bind TIGIT^+^ T cells, impairing CD8^+^ T cell cytotoxicity and boosting Treg functions, and the last one comprising HLA-E^+^ TAMs and CD8^+^ T cells (122). A similar neighborhood between SPP1^+^ TAMs and exhausted CD8^+^ T cells, mediated by GDF15 and TGFBR2, was also described in metastatic gastric cancer, where it was linked to the induction of immunosuppression (123). In esophageal squamous cell carcinoma a subset of TREM2^+^ TAMs was shown to interact with CD8^+^ TEX cells or Tregs through SPP1-related signaling pathways including SPP1-CD44 and SPP1-(ITGA4^+^ITGB1) (124). SPP1 and TREM2 expression also characterize TAMs found to interact with CD8^+^ TEX cells in lymph node metastases of head and neck squamous cell carcinoma (HNSCC), where three main ligand-receptors pairs emerged: SPP1-CD44, LGALS9-HAVCR2 (TIM-3) and ALCAM-CD6 (125). Another recent study identified two pro-tumoral hubs in CRC by means of spatial transcriptomics, one including SPP1^+^ TAMs, enriched for coagulation and cholesterol homeostasis pathways, that communicate with CD8^+^ T cells primarily via the inhibitory SPP1-CD44 axis, and another involving SELENOP^+^ TAMs, enriched for inflammatory responses and TNF signaling, interacting with T cells via the B2M-KLRD1 axis, that can similarly inhibit T cell cytotoxicity (126). Notably, before the advent of high-dimensional omics, our group had described close interactions between CD206^+^CD163^+^ TAMs and CD8^+^ T cells in the stromal regions of squamous cell human NSCLC and murine breast cancer that limited the migration and infiltration of T cells into tumor nests through physical trapping. In mice, these stromal hubs were linked to tumor progression and resistance to immunotherapy and their disruption using a CSF1R inhibitor unleashed T cell migration and the anti-PD-1 therapeutic potential (12).

##### 3.1.1.2 Hypoxic niches

Several authors reported the presence of immunosuppressive TAM-TIL niches in hypoxic regions of tumors. In human and murine glioblastoma (GBM), CD206^+^ARG1^+^ TAMs are co-sequestered in these areas, likely by means of CCL8 and IL-1β, with exhausted CD8^+^ TILs expressing TIM-3 and PD-1. The presence of such hypoxic TAM-TIL niche positively correlates with higher glioma grade and the aggressive mesenchymal transcriptional subtype of GBM, strongly linked to immunosuppression and poor patient outcome (99). In mouse models of melanoma and breast cancer, Kersten and colleagues evidenced a similar hypoxic niche composed of TAMs with a metabolically altered state, marked by high expression of lipid/scavenger genes like *Apoe* and *Ms4a7*, and exhausted CD8^+^ T cells, that seem to protect rather than eliminate the tumor (98).

##### 3.1.1.3 TAM-Treg niches

In primary breast tumors, a CD169^+^ TAM subset seems to be associated with TLLS and, specifically, with Treg and Breg infiltration, being an independent negative prognostic factor. Surprisingly, the same CD169^+^ TAM-TLLS niche in lymph node metastases of these tumors correlated with improved prognosis (90).

In CRC, an immunosuppressive and pro-tumorigenic hub was described together with an anti-tumor (ISG)/CXCL13 immune hub. In this niche, mainly found at the interface between the tumor and the luminal margin, especially in highly necrotic regions, monocytes and macrophages recruit additional myeloid cells, including neutrophils, and interact with Tregs and IL-17^+^ T cells. The amplification of inflammation and immune cell recruitment is further reinforced by non-immune cells like tumor cells and CAFs (127).

Alongside traditional TLS-like phenotypes, an immunosuppressive microenvironment named *Macrophage/T-Clustered* has been identified in clear cell renal cell carcinoma (ccRCC), characterized by an abundance of CD163^+^ macrophages, Tregs and CD8^+^ T cells, but a distinct absence of B cells. The authors speculate that the physical interactions between TAMs and TILs create dense cell clusters that drive T cell exhaustion, contributing to the immunosuppression of the TME. Among the different phenotypes, this one was associated with the poorest survival and treatment failure (128).

While in squamous cell NSCLC the cellular neighbors of TAMs are preferentially cancer cells and neutrophils, in lung adenocarcinoma CD163^-^ TAMs have been shown to interact with B cells, Tregs and CD163^+^ macrophages. Among the different cell clusters identified in these tumors, only these niches are associated with worse patient survival (130).

In HNSCC, specialized cellular neighborhoods termed TLS2 have been identified as immunosuppressive hubs. These structures are characterized by the co-localization of CD163^+^ TAMs and Tregs alongside conventional CD4^+^ and CD8^+^ T cells, polymorphonuclear myeloid-derived suppressor cells (PMN-MDSCs), and associated vasculature (131).

##### 3.1.1.4 Intraepithelial immunoregulatory niches

In NSCLC, a niche comprising CD68^+^CD163^+^ macrophages interacting with Tregs and T cells, enriched in metabolic immune regulators (IDO1, ARG1) and a wide array of immune checkpoint molecules (PD-L1, PD-L2, CTLA-4, B7-H3, OX40L) and associated with immunosuppression, has been described inside tumor nests (132). While the presence of intraepithelial T cells is generally associated with a better clinical outcome, in these hubs the function of effector T cells might be locally suppressed through interactions with Tregs and macrophages. This underscores the importance of mapping the cellular neighborhood of intraepithelial T cells to better understand their functional state and the resulting anti-tumor immune outcome.

#### 3.1.2 TAM-TIL niches associated with good prognosis/response to ICB

##### 3.1.2.1 Inflammatory chemokine-driven TAM-TIL hubs

Several studies have identified that TAM-TIL niches regulated by chemoattractant axes in human tumors are generally associated with a positive clinical outcome and response to ICB. The most frequent axis in these hubs involves the expression of CXCL9/CXCL10/CXCL11 by TAMs, and CXCR3 by T cells, although other chemokine-receptor pairs have recently been described. In lung cancer, *stem-immunity hubs* (named after the enrichment of stem-like TCF7^+^PD-1^+^CD8^+^ T cells) were described in stromal regions, often at the peritumoral edge, where they were associated with response to immunotherapy (133). Within these hubs, two axes of myeloid-T cell interactions were observed, one consisting of mReg DCs (expressing CCR7, IDO1 and LAMP3) interacting with Treg cells/CD4^+^ T cells through the CCL17/CCL22-CCR4 and CCL19-CCR7 axes, and one composed of CXCL10^+^ macrophages interacting with TCF7^−^CD8^+^ T cells, likely through the CXCL9/CXCL10/CXCL11-CXCR3 axis (133).

Another study recently described a niche between pro-inflammatory TAMs expressing CXCL9 and CXCL11 and TEX expressing CXCR3 that correlates with response to ICB and improved survival across different cancer types (134). Another immune hub involving CXCL9/CXCL10/CXCL11^+^ TAMs and cytotoxic CD8^+^ T cells has recently been described by spatial transcriptomics in CRC along with more immunosuppressive TAM clusters, further highlighting the complexity of the TME where anti-tumoral and pro-tumoral immune niches may coexist in the same tumor (126).

In CRC cancer, multiple immune hubs have been described. Among them, an interferon-stimulated gene *(ISG)/CXCL13 immune hub*, particularly enriched in DNA mismatch repair deficient (dMMR) tumors, shows interactions between activated IFN-γ^+^ and CXCL13^+^ T cells and CXCL10/CXCL11^+^ myeloid and malignant cells. This hub forms foci throughout the tumor center (127). Even though the authors have not formally explored the relationship between the presence of this hub and prognosis or response to immunotherapy of CRC patients, IFN-γ-profiles and CXCL13 expression have already been linked to ICB response in other studies (138). Moreover, the enrichment of this hub in dMMR tumors, known to be immunogenic and responsive to immunotherapy, strongly suggests that it might represent a relevant predictive biomarker for ICB. In another study spanning across 9 cancer types, two types of immune hubs were described: *TLS-hubs*, containing IFN-macrophages (CXCL10^+^STAT1^+^) with CD4^+^ TFh cells, differentiated B cells, together with mQuiescDCs, a mature, quiescent dendritic cell subset that functionally interacts with T and B cells to regulate local immunity. In these hubs, CD4^+^ TFh cells interact with IFN-TAMs through the PD-1-PD-L1 axis and, in addition, with DCs through the CCL19-CCR7 axis. The second type of hub, *Type 1 immunity hub*, is composed of early/inflammatory macrophages (CCR2^+^ Mono-like Macs), IL1β^+^CCL3/4^+^PD-L1^+^ Inflam TAMs, neutrophils, and CXCL1^+^CXCL3^+^CCL2^+^ lipid-associated macrophages (LAM2s), immune-regulatory cells (mRegDCs and CD4^+^ Tregs), as well as AXL DCs, lymphatic endothelial cells (ECs), and PD-1^+^ T cells (CD4^+^Th1, CD8^+^TEX and proliferating T cells). In these hubs, TAM-TIL interactions likely involve TNF and IFN-γ signaling, as CD4^+^ Th1-cells and other T cell subtypes express TNF and IFN-γ, while macrophages and DCs express their receptors (TNFRSF1A/B and IFNGR1/2). Both *TLS hubs* and *Type 1 immunity hubs* were found to correlate with T cell reactivity and response to ICB in breast cancer and HNSCC (135).

##### 3.1.2.2 Antigen-presenting C1QC^+^ TAM-TIL hubs

In CRC, Zhang and colleagues identified a specific niche composed of C1QC^+^ RTMs and CD4^+^ T cells, where interactions seem primarily driven by MHC-II-antigen presentation (136). These hubs are more frequent in anti-PD-1 responders, regardless of microsatellite status. Moreover, the physical proximity between these cells predicts clinical outcome, with shorter median distance between C1QC^+^ RTMs and CD4^+^ T cells correlating with a greater survival benefit. This spatial relationship appears functionally relevant, as CD4^+^ T cells located in close proximity to C1QC^+^ RTMs show higher expression of activation markers compared to more distant cells. C1QC^+^ RTMs in these niches exhibit strong antigen presenting ability and low immunosuppressive features.

##### 3.1.2.3 FOLR2^+^-TIL hubs

Two different FOLR2^+^ TAM-TIL niches with favorable impact on patient outcome have been recently described. In the first one, described in the stromal compartment of breast cancer, especially in perivascular regions, FOLR2^+^ TAMs interact with CD8^+^ T cells and seem to prime them, functioning as APCs (72). *FOLR2* expression was associated with genes controlling cytotoxic T cell function (*GZMA, GZMB, GZMK, PFR1, KLRB1*, and *KLRD1*) but not with genes related to T cell dysfunction like *LAG3.* Moreover, a high percentage of CD8^+^ T cells in close contact with FOLR2^+^ macrophages was associated with a better outcome in these patients, whereas no such association was observed for CD8^+^ T cells interacting with TREM2^+^ macrophages (72). Matusiak and colleagues also described a similar neighborhood containing FOLR2^+^ RTMs with CD4^+^ T cells, and CD8^+^ T cells, but also Tregs and DCs, located in the peritumoral stroma as well as in benign tissue of both breast and colon cancer (83).

A different niche containing FOLR2^+^ TAMs has been described inside TLSs of different human carcinomas. FOLR2^+^ TAMs express TIM-4 and are found in T cell zones of TLSs. Immunogenic cancers, like microsatellite instability (MSI) CRC or lung cancer, seems to be enriched in TIM-4^+^FOLR2^+^-containing TLSs and their density directly correlates with the density of infiltrating CD8^+^ T cells, suggesting a positive role for anti-tumor immunity (103).

##### 3.1.2.4 Intraepithelial PD-L1^+^ TAM-TIL hubs

In high-grade serous epithelial ovarian cancer (HGSOC), intraepithelial PD-1^+^CD8^+^ TILs have been found to interact with PD-L1^+^CD11c^+^ cells, comprising DCs and CD68^+^ macrophages, within tumor islets. The presence of high densities of both intraepithelial PD-1^+^CD8^+^ TILs and PD-L1^+^CD11c^+^ myeloid cells is associated with improved survival in these patients (137). In a similar way, the presence of CD68^+^CD206^-^ macrophages and PD-L1^+^ cells in the epithelial compartment of fully inflamed breast tumors has also been positively associated with the presence of CD8^+^ TILs and good patient outcome (48).

##### 3.1.2.5 TLS hubs

In addition to the previously mentioned *TLS-hubs* described by Lodi and colleagues (135) in several solid tumors, TLSs generally contain TILs and myeloid cells. Although myeloid DC-LAMP^+^ DCs have been frequently described within these structures, macrophages are also present, albeit less well characterized.

In ccRCC, a *TLS-like phenotype* was evidenced, characterized by the presence of CD8^+^ T cells (including Granzyme B^+^ T cells), CD163^−^ macrophages and B cells, and associated with better survival compared to the other phenotype found in these tumors, the *macrophage/T-clustered phenotype* (described below) (128).

The presence of TLSs containing TIM-4^+^FOLR2^+^ macrophages that contact CD3^+^ T cells has also been correlated with good prognosis across several cancers, although the specific T cell phenotypes have not been explored in this context (103). Another study evidenced the presence of CD163^+^ TAMs and Tregs in both intratumoral and peritumoral TLSs of CRC liver metastases; in this case, however, the spatial localization of TLSs rather than their cellular composition seems to be relevant for prognosis, as patients with a higher proportion of intratumoral compared to peritumoral TLSs experience a better clinical outcome (129). In HNSCC, a multiparametric proteomic study revealed TLS hubs, called TLS1, where CD4^+^ and CD8^+^ T cells interact with Ig-producing plasma cells but also with CD68^+^CD163^-^ M1 TAMs, monocytes and neutrophils (131).

### 3.2 Functional impact of TAM-TIL hubs

**Figure 2:**
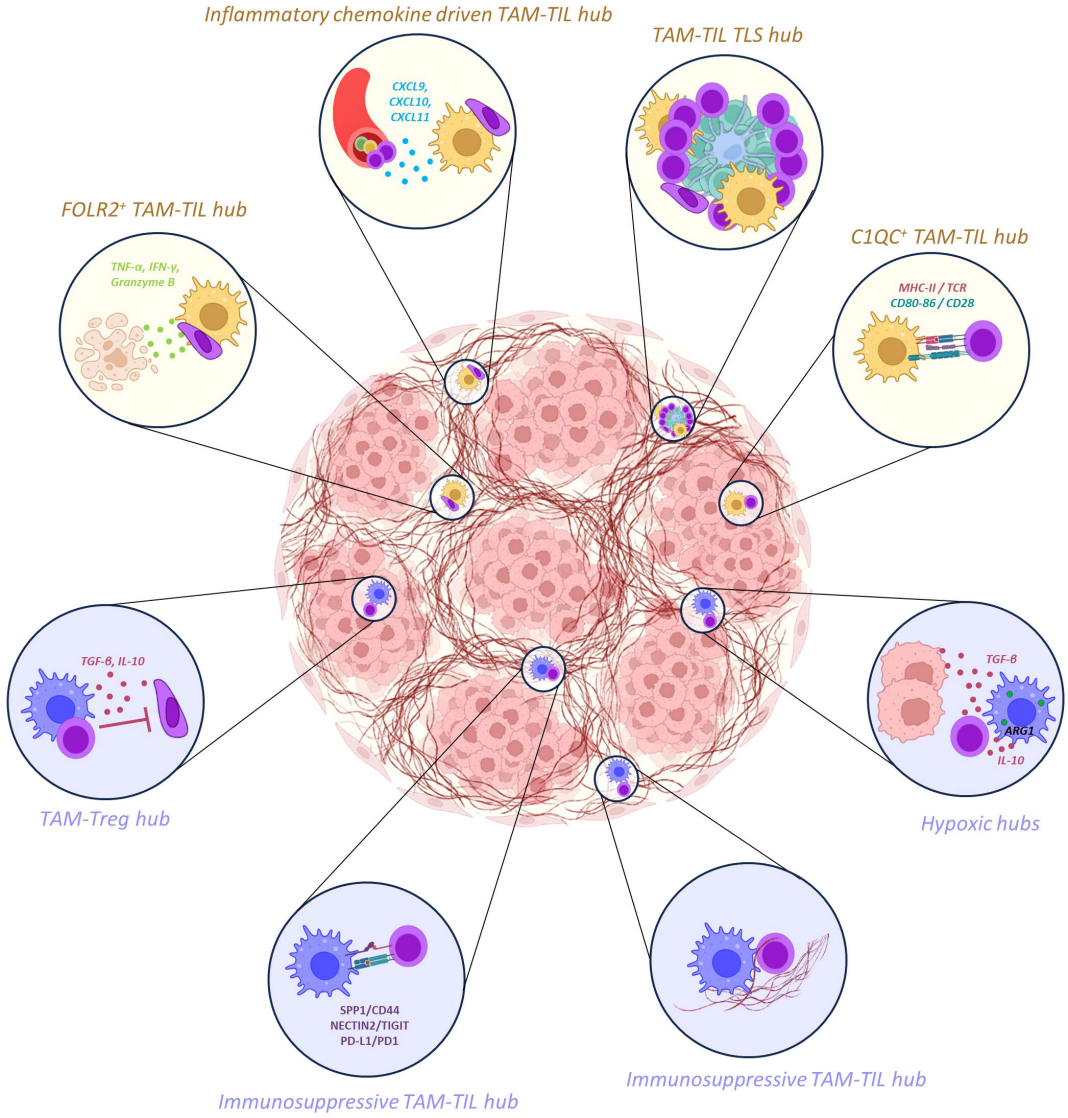
Overview of different types of several dominant anti-tumoral or pro-tumoral TIL/TAM-TIL niches. We provide eight examples of TAM-TIL hubs with either anti-tumoral (yellow background) or pro-tumoral (blue background) impact. The specific cell phenotypes and the mechanisms of activation/suppression taking place within each hub are detailed in the zoomed circles. Four types of anti-tumoral niches are described on the upper part of the **Figure 1**) *FOLR2^+^ TAM-TIL hubs*, containing highly cytotoxic T cells expressing IFNγ, TNFα and granzyme B; 2) *inflammatory chemokine-driven TAM-TIL hubs*, where inflammatory chemokines produced by TAMs, such as CXCL9, CXCL10 and CXCL11, lead to the homing of TILs in the TME; 3) *TLS hubs*, where T cells interact with several cell types including TAMs; 4) *antigen-presenting TAM-TIL hubs*, harboring C1QC^+^ TAMs that perform MHC II antigen presentation and costimulation and associate closely to CD4^+^ TILs. Four types of pro-tumoral niches are described on the lower part of the **Figure 1**) *TAM-Treg hubs*, where effector T cells can be functionally inhibited by both TAMs and Tregs; *immunosuppressive TAM-TIL hubs*, either comprising 2) inhibitory crosstalk through the SPP1/CD44, Nectin2/TIGIT and PD-L1/PD-1 axes or 3) hubs where TAMs trap TILs in the stroma through non-productive interactions, leading to T cell immune exclusion; 4) *hypoxic hubs*, where TAMs can induce T cell exhaustion by means of ARG1 metabolism and the release of inhibitory soluble factors, such as TGF-β and IL-10. Created with BioRender.com.

The transition from spatial mapping to functional interpretation reveals that the clinical impact of TAM-TIL niches is fundamentally driven by the molecular crosstalk established within these hubs. Rather than acting as independent entities, TAMs and TILs function as a synchronized regulatory unit, where their reciprocal interactions lead to the formation of either anti-tumoral (immune-active) hubs or pro-tumoral (immunosuppressive) hubs (**Figure 2**). We detail below the main mechanisms of TAM-TIL crosstalk present in both types of niches.

#### 3.2.1 “Bad” neighborhoods: pro-tumoral niches

##### 3.2.1.1 Contact-dependent inhibition of TIL activities

TAM-mediated immunosuppression in pro-tumoral niches is largely due to their ability to inhibit T cells. TAMs that express lower levels of MHC class II and costimulatory molecules lose their antigen-presenting capacity, thereby limiting the initiation of the anti-tumor adaptive response (139, 140). In the B16-OVA melanoma model, Kersten and colleagues demonstrated that TAMs engage in prolonged synaptic interactions with tumor-antigen-specific OT-I CD8^+^ T cells. TAMs trigger only weak TCR stimulation, leading to the upregulation of the transcription factor IRF4 in T cells, which initiates and sustains an epigenetic program of terminal T cell exhaustion marked by high TOX, PD-1, and CTLA-4 expression. These TOX^+^PD-1^+^CTLA-4^+^ CD8^+^ T cells ultimately secrete M-CSF, promoting monocyte recruitment, and correlate with macrophage abundance in the TME, suggesting the maintenance of a highly immunosuppressive environment (98).

A major axis inducing T cell exhaustion, exploited by ICB and now well recognized, involves PD-1 on T cells and its ligands (PD-L1, PD-L2) on TAMs (132, 134, 141). Despite the inhibition mediated by this axis, PD-L1 expression is often an “adaptive resistance” mechanism upregulated in response to T cell-derived IFN-γ. Consequently, while PD-L1 characterizes pro-tumoral niches, its presence underscores an active anti-tumor response and identifies immunosuppressive barriers that are fundamentally reversible through immune checkpoint blockade. Another mechanism that reduces co-stimulation involves the inhibitory receptor CTLA-4, expressed by experienced T cells. The engagement of CD80/CD86 by CTLA-4^high^ Tregs in the local microenvironment may also impair adequate restimulation of effector T cells (142).

Many members of the B7 superfamily other than PD-L1 and PD-L2 are upregulated by TAMs within the TME and are now recognized as negative regulators of T cell functions, such as B7-H3, B7-H4 and B7-H5 (VISTA) (143, 144).

Non-classical MHC molecules expressed by TAMs, such as HLA-E, HLA-F and HLA-G, have also been shown to interact with inhibitory receptors on cytotoxic T cells and NK cells, thereby inhibiting their activation (145, 146). In NSCLC, tumor and myeloid cells upregulate HLA-E, which binds to CD8B on T cells and the inhibitory NKG2A receptor on NK cells to directly impair their cytotoxicity (122). In the same study, the NECTIN2-TIGIT axis also emerged for inhibitory TAM-TIL crosstalk, with NECTIN2 expressed by TAMs binding to the inhibitory receptor TIGIT on T cells and NK cells, reducing their proliferation and effector function while simultaneously boosting the suppressive capabilities of Tregs (122). This axis is increasingly recognized as relevant for therapeutic targeting to reduce immunosuppression in the TME and improve immunotherapy efficacy (147, 148).

Another molecule that has been frequently involved in TAM-mediated T cell suppression in the last decade is TREM2 (149). TREM2^+^ TAMs secrete significantly low levels of CXCL9 and high levels of galectin-1, IL-10 and TGF-β, known to suppress T cell activity (101, 150)(see next paragraph). Although the binding of TREM2 to its ligands (such as amino-phospholipids on apoptotic cells) triggers a signaling cascade that reprogram macrophages into an anti-inflammatory state, with reduced production of reactive oxygen species and pro-inflammatory cytokines and increased secretion of immunosuppressive factors, the impact of the direct binding of TREM2 on TAMs with TREM2 ligands on T cells currently remains unclear (61).

##### 3.2.1.2 Production of soluble factors with immunosuppressive effects

TAMs contribute to the establishment of immunosuppressive niches within the TME through the localized production of cytokines and chemokines. In particular, TAM-derived IL-10 and TGF-β suppress cytotoxic CD8^+^ T cell and Th1 responses while promoting the accumulation and stability of Tregs within macrophage-rich tumor regions (47, 81, 103, 151, 152). These cytokine gradients may therefore contribute to the formation of spatially restricted immunosuppressive hubs in which effector lymphocytes become functionally restrained. TGF-β may nevertheless exert beneficial effects on T cells by promoting their differentiation into TRM cells, highlighting the context-dependent impact of TAM-derived cytokines on anti-tumor immunity.

In many of the aforementioned studies, pro-tumoral hubs are characterized by the release of SPP1 by TAMs. TAM-derived SPP1 binds CD44 on T cells, restricting T cell infiltration while promoting dysfunction. In parallel, SPP1 can autocrinally induce PD-L1, CD73, IL-10, and TGF-β expression in macrophages, thereby further reinforcing local immunosuppression (101, 123, 126, 153). SPP1^+^ TAMs also secrete GDF15, which binds TGFBR2 on exhausted CD8^+^ T cells, enhancing inhibitory receptor expression, promoting apoptosis, and limiting T cell extravasation and infiltration (123, 154).

Additional TAM-derived soluble mediators also contribute to T cell exclusion. For example, Galectin-1 induces PD-L1 expression by endothelial cells, creating a vascular barrier that limits CD8^+^ T-cell recruitment into tumors (101, 150). TAMs also exploit the CD39/CD73 ectonucleotidase axis to convert immunostimulatory ATP into adenosine, thereby reinforcing T-cell exhaustion (155). Finally, the production of CCL22, CCL2, and CCL3 within pro-tumoral hubs promotes local Treg recruitment and accumulation (156).

##### 3.2.1.3 Physical trapping that prevents T cell infiltration into tumor nests

Within the tumor microenvironment, TAMs can act as a physical barrier that prevents T cell infiltration into tumor nests. In human NSCLC, we previously demonstrated that TAMs engage in long-lasting contacts with CD8^+^ T cells in the stroma, effectively trapping them and hindering their capacity to migrate. We observed the same physical trapping in a murine breast cancer model, where depleting TAMs through CSF-1R inhibition released this brake, enhancing the ability of T cells to migrate, reach tumor cells, and ultimately improve the efficacy of anti-PD-1 therapy in otherwise resistant tumors (12). Consistently, several independent studies have also shown an increase in intra-tumoral CD8^+^ T cells following targeting of the CSF-1/CSF-1R pathway, supporting the notion that T cell migration and localization are heavily regulated by TAMs (12, 157, 158).

##### 3.2.1.4 Metabolic immunosuppression

TAMs are capable of degrading amino acids present in the environment through the expression of various amino-acid catabolizing enzymes (e.g. iNOS, ARG-1, IDO1 and IL4I1). Since T cells are auxotrophic (i.e. unable to synthetize) for most amino acids, the reduction of the concentration of notably arginine, tryptophan, and phenylalanine in the TME represents another checkpoint for their proliferation and function (159, 160). Furthermore, the catabolites released following amino acid degradation also interact with cells in the TME and subvert the protective anti-tumor response.

Arginase-1 hydrolyzes arginine to ornithine, starving T cells of arginine needed for the expression of the TCR CD3ζ (zeta) chain and therefore for TCR signaling and proliferation. On the other hand, the metabolism of arginine through iNOS produces nitric oxide (NO), that can inhibit signal transduction pathways in T cells, and peroxynitrites, that can physically disrupt the interaction between the TCR and MHC molecules on APC or nitrosylate T cell attracting chemokines (152, 161). TAMs also express the enzymes indoleamine-2,3-dioxygenase (IDO) and tryptophan-2,3-dioxygenase (TDO or TDO2), which catabolize the essential amino acid tryptophan into kynurenine (162). Similar to arginine, tryptophan starvation downregulates the TCR CD3 ζ-chain, halting T cell proliferation and cytotoxicity. In addition, the accumulated kynurenine binds to the aryl hydrocarbon receptor (AhR) on T cells, which induces Treg differentiation and negatively regulates CD8^+^ T cell function (152, 156). In a similar way, IL4I1 expressed by TAMs oxidatively deaminates aromatic amino acids, mainly L−phenylalanine, tryptophan, and tyrosine (163). Upon tryptophan degradation, IL4I1 generates kynurenic acid and indole metabolites that activate AhR, thereby exerting the previously mentioned immunosuppressive effects (81, 95, 164). Notably, our group showed that macrophage-specific IL4I1 inactivation in melanoma models reduced tumor growth. This benefit was associated with enhanced anti-tumor CD8^+^ T cell functions and the reprogramming of TAMs toward an anti-tumor phenotype (165).

TAMs can also exploit lipid metabolism to mediate immunosuppression, for instance by utilizing accumulated intracellular cholesterol to synthesize and release glucocorticoids that promote CD8^+^ T cell dysfunction and contribute to resistance to immunotherapy (152). TAMs also metabolize arachidonic acid via the cyclooxygenase-2 (COX-2) enzyme producing eicosanoids, such as PGE2, that actively suppress CD8^+^ T cell responses and promote the reprogramming of TAMs toward a regulatory phenotype (152, 156, 166).

Moreover, TAMs are highly metabolically active and consume glucose, thereby limiting nutrients availability for T cells and impairing their survival, proliferation, and anti-tumor effector functions (152, 156).

#### 3.2.2 “Good” neighborhoods: anti-tumoral microdomains

##### 3.2.2.1 Antigen presentation and costimulation

TAMs that highly express MHC Class I and II molecules alongside antigen-processing genes can facilitate direct and robust restimulation of T cells within the TME. TAMs also deliver costimulatory signals through CD80-CD86/CD28, and OX40L/OX40 interactions, and secrete cytokines that activate cytotoxic T cells, such as IL-12p70, TNF-α, and type I IFN (as described in the last paragraph of this section). In metastatic CRC, C1QC^+^ RTMs primarily function as APCs through MHC-II signaling and their physical interaction with CD4^+^ T cells is associated with improved immunotherapy response (136). In addition, previously mentioned resident mammary FOLR2^+^ macrophages, which correlate with improved clinical outcome, have a transcriptional signature supporting antigen presentation to TILs in breast cancer patients (72).

TAMs can also maintain a functional pool of anti-tumor T cells via tumor antigen cross-presentation, with evidence mainly deriving from murine models.

Mechanistic insights from murine B16F10-OVA melanoma models demonstrate that CD206^hi^ TAMs cross-present tumor antigens, with an efficiency comparable to that of cDC1, and trigger T cell IFN-γ production *in vitro* (167). The ability of specific macrophage subsets to cross-present antigens has also been demonstrated in humans, notably in settings where monocyte-derived macrophages elicited a greater IFN-γ production by a MelanA-specific CD8^+^ T cell clone than monocyte-derived dendritic cells (168). Interestingly, a population of CD206^hi^ TAMs, associated with better patient prognosis, has been described in melanoma patients, further supporting the evidence that cross-presentation mediated by TAMs can take place in human tumors (167). Similar TAMs are also able to provide costimulatory signals to anti-tumor T cells within TAM-TIL niches. This interaction may be particularly important in light of recent work suggesting that the delivery of a second activation signal, supported by costimulation, must occur within the TME rather than in draining lymph nodes to enable full differentiation of effector CD8^+^ T cells (169). In ovarian cancer, CD8^+^ T cells located within niches enriched in macrophages and DCs displayed superior effector functions compared with those positioned farther away from APCs. Engagement of CD28 was required *in situ* to maintain their effector functions and to support their activation in response to anti-PD-1-based immunotherapy (137).

##### 3.2.2.2 T cell recruitment through chemokines

TAMs can act at multiple levels for the recruitment of immune cells, particularly of cytotoxic T cells. When properly activated, their NO production induces the expression of adhesion molecules by endothelial cells, thereby enabling T cell diapedesis (170). Moreover, as described above, TAMs in anti-tumoral hubs frequently produce chemokines, such as CXCL9 and CXCL10, responsible for the recruitment of T cells to the tumor site (152, 156, 166).

##### 3.2.2.3 Reciprocal TAM-TIL activation through cytokines

In anti-tumoral niches, TAMs and TILs produce cytokines and can reciprocally activate each other. In human and mouse gliomas, the presence of CD169^+^ macrophages are associated with improved recruitment of CD8^+^ T cells and NK cells in the TME. Using IFN-γ^-/-^ mice, the authors demonstrated that the differentiation of monocytes into CD169^+^ TAMs depends on IFN-γ derived from NK cells and CD8^+^ TILs, highlighting positive crosstalk between immune effectors and macrophages (171). In a similar way, type 1 immunity hubs described across different human tumors and associated with response to ICB rely on TIL-TAM cross-activation mediated by IFN-γ and TNF-α (135).

Ultimately, mapping the functional diversity of TAM-TIL niches is essential for designing targeted interventions capable of remodeling these hubs shifting the local TME from immune evasion toward active tumor rejection.

## 4 Therapy-driven remodeling of TAM-TIL niches

Therapeutic interventions, whether targeting macrophages directly or revitalizing T cells via ICB, trigger a cascade of functional and spatial reprogramming. While these agents are mainly designed to be cell-centric, their clinical efficacy is ultimately realized at the level of the TAM-TIL functional and biophysical interface. In this section, we analyze some examples of how perturbing the myeloid compartment remodels the spatial architecture of TAM-TIL niches, restoring T cell activity. Conversely, we explore how successful ICB induces a T cell-derived cytokine burst that drives the differentiation of anti-tumor macrophages. Together, these processes illustrate how therapeutic efficacy depends on the functional reorganization of the niche, converting suppressive barriers into optimized immune hubs.

### 4.1 Therapeutic shaping of TIL-TAM functional interplay

#### 4.1.1 Blockade of monocyte recruitment (CCR2/CCR5 Axis)

The CCL2-CCR2 axis serves as the principal pathway for classical monocyte mobilization into the tumor, where they typically develop into suppressive TAMs during tumor progression. Blocking this axis was initially viewed as a way to prevent the myeloid pool from replenishing itself. Moreover, monocyte depletion removes CCL22/IL-10-mediated Treg support and direct suppression, reactivating effector CD8^+^ T cells (172). Nevertheless, accumulating evidence indicates that the recruitment of monocyte-derived populations can be essential for tumor immunosurveillance and immunotherapy efficacy, specifically when they differentiate into anti-tumoral or antigen-presenting cells rather than suppressive TAMs (32, 94, 173–176). This underscores the importance of selectively targeting suppressive myeloid populations rather than broadly inhibiting myeloid cell infiltration. Beyond CCR2, the CCR5 receptor has also been targeted. CCR5 inhibition effectively disrupts the suppressive myeloid axis, but it is also required for Th1 effector cell homing, so its wide pharmacological blockade poses the risk of concurrently excluding cytotoxic T cells essential for treatment efficacy (177, 178). Since these chemokine axes are pleiotropic and influence multiple diverse immune populations and functions, systemic blockade may lead to unintended immune suppression. To mitigate this, targeted strategies toward myeloid cells and their precursors are necessary to preserve the functionality of the lymphoid compartment while selectively disrupting the suppressive myeloid axis (179).

#### 4.1.2 Targeting the CSF1/CSF1R axis

The CSF1R pathway is a major survival, proliferative and differentiative regulator in myeloid lineage cells. Therapies targeting this pathway generally operate through two distinct, but rather complementary, mechanisms: depletion and reprogramming. Blockade of CSF1R disrupts survival signals, mainly through PI3K/Akt pathways, leading to the depletion of TAMs, particularly those with suppressive functions, like the CD206^high^ TAMs (158, 180). While CSF1R is a broad myeloid regulator, its role is most critical for monocyte-derived macrophages; in contrast, neutrophils and conventional DCs primarily rely on G-CSF and FLT3L, respectively, making them less sensitive to CSFR1 inhibition. Moreover, this inhibition sensitizes the residual TAMs to pro-inflammatory reprogramming (158, 181). The function of these surviving cells extends beyond distal signaling, being essential for establishing spatial niches that recruit T cells mainly via CXCL9/10 production (182). More importantly, they support inflammation by upregulating anti-tumor immunity genes, such as *Il12a*, *Ifna, Ifnb1, Ifng, Cxcl10*, and *Nos2*, and might also physically engage these T cells for antigen presentation by upregulating the expression of MHC molecules (158). This remodeling has been associated with robust CD8^+^ T cell infiltration in several studies (158, 182), thereby promoting T cell reactivation when combined with other therapeutic strategies, particularly immune checkpoint inhibitors (12). In addition, the use of covalent CSF1R inhibitors may promote durable expansion of tumor antigen-specific CD8^+^ T cells and improved long-term tumor control, by preventing the rapid repopulation of suppressive TAMs during dosing intervals (180, 183).

Interestingly, clinical translation revealed that emactuzumab (anti-CSF1R) combined with atezolizumab (anti-PD-L1) dosing requires biological optimization over dose maximation. Phase I trials indicated that objective responses correlated with increased density of activated CD8^+^ TILs. However, these studies identified a high-dose paradox. Higher doses resulted in greater total macrophage depletion, but not necessarily improved T cell infiltration and responses (184). Indeed, total ablation of the CSF1R^+^ compartment can be counterproductive, as it eliminates not only suppressive cells but also the myeloid population required to initiate anti-tumor immunity, as demonstrated by studies showing that the absence of macrophages can actually decrease the efficacy of certain immunotherapies (185, 186). These findings support the notion that complete ablation of the macrophage compartment, rather than its reprogramming, proves detrimental, as it destroys the cellular infrastructure required for T cell positioning and reactivation. Therefore, an Optimal Biological Dose (OBD) outperforms the traditional Maximum Tolerated Dose (MTD) by preserving this supporting myeloid scaffold while still reducing suppressive TAMs. By intentionally favoring reprogramming over total clearance, the success of OBD highlights that therapeutic efficacy relies on maintaining direct spatial interactions with residual myeloid partners, not by simply eliminating suppression (184, 187).

#### 4.1.3 Molecular reprogramming via PI3Ky inhibition

Strategies focusing on the intracellular pathways governing macrophage functions may constitute an alternative to preserve spatial niches favoring TIL reactivation. In the TME, PI3Kγ activates C/EBPβ and inhibits NF-κB in TAMs. This signaling architecture drives a transcriptional program typical of suppressive TAMs, including the production of *Arg1, Il10*, and other suppressive mediators (188). Pharmacological inhibition of PI3Kγ effectively relieves NF-κB suppression and decreases C/EBPβ activity, restoring the production of pro-inflammatory cytokines (e.g., IL-12) while downregulating suppressive programs (*Arg1, Il10).* This rewires TAMs from tumor supporters to immune effectors (188, 189).

The downstream effect of this molecular switch is a significant modification of the adaptive immune landscape. Indeed, blocking PI3Kγ increases the recruitment and retention of effector T cells by promoting an inflammatory chemokine environment, specifically through NF-κB-dependent induction of CXCL9 and CXCL10 (182, 189). Clinical data from studies of the PI3K inhibitor eganelisib (IPI-549) indicate that this effect is not determined by the absolute quantity of infiltrating lymphocytes, but by their functional quality. The therapy enables a myeloid-to-T cell handoff, in which reactivated antigen-presenting niches effectively boost T celchuls after ICB (182). However, emerging single-cell analyses indicate that CD8^+^ T cell density alone does not guarantee efficacy (190); instead, the spatial proximity of non-exhausted T cells to these reprogrammed myeloid niches appears to be the key determinant of response to therapy (187), underscoring that PI3Kγ inhibition restores the necessary spatial architecture for durable anti-tumor immunity.

#### 4.1.4 Beyond TIL reactivation following ICB

Although the primary targets of ICB therapies are anti-tumor T cells, high-dimensional single-cell RNA sequencing analyses have revealed that therapeutic success, i.e. tumor regression, results from profound remodeling of the TME, including repolarization of TAMs. The primary driver of this reprogramming appears to be the revitalized T cell compartment, which acts as a cytokine trigger. Gubin and colleagues found that effective ICB, specifically with anti-PD-1 and anti-CTLA-4, leads to fast proliferation of activated T cells and high levels of IFN-γ secretion (191). This mechanistic reliance on INF-γ has been validated in human patients, where transcriptomic profiling has identified an INF-γ gene signature as a superior predictor of response to PD-1 blockade across various solid tumors compared to tumor mutational burden or PD-L1 expression alone (192). Successful therapeutic responses require intact INF-γ signaling in tumor cells, since loss-of-function mutations in genes of this pathway, including JAK1/2 or STAT1, have been demonstrated to drive primary resistance to ICB (14, 193). However, beyond this direct effect on tumor cells, this cytokine burst serves as an important differentiation checkpoint for TAMs. The identity of these regression-associated macrophages has been further refined by recent single-cell transcriptomic studies linking the earlier molecular definitions to the broader concept of multicellular organization. Notably, the iNOS^+^ population highlighted in murine models of successful ICB (191) significantly overlaps with a distinct human macrophage subset enriched for CXCL9, CXCL10, and MHC-II gene signatures in biopsies of ICB responders at baseline (5, 194). However, the identification of these CXCL9^+^ TAMs gain true functional significance only when viewed through a spatial, or “geographical,” perspective.

### 4.2 Consequences of therapeutic interventions on TAM-TIL niche spatial organization

While functional reprogramming induced at the single-cell level is essential, the spatial distribution of immune cells within the TME emerges as an equally crucial parameter governing therapeutic efficacy (187). Some preclinical studies already helped envision the dynamic changes that may occur in TAM-TIL niches after therapy, whereby the initial localization of immune cells initiates signaling events in neighboring cells, leading to their elimination or migration and ultimately driving continuous cellular redistribution and the emergence of new intercellular interactions (158).

One example refers to perivascular TAMs. The vasculature is usually altered in the TME and represents an important physical and functional barrier for T cell infiltration (195). Interestingly, the accumulation of iNOS^+^ TAMs, found in both human and mouse models following treatments like low-dose radiation or TLR-9 activation, has been associated with the normalization of tumor vessel size and permeability (170, 196). Mechanistically, this normalization relies on a dual action: the downregulation of pro-angiogenic factors, such as VEGF and GM-CSF, together with the production of NO by TAMs. This NO signaling is critical for the upregulation of VCAM-1 on endothelial cells, effectively transforming the vessel wall from a barrier into a gateway that actively supports T cell adhesion and homing.

Furthermore, therapeutic intervention such as CSF1R inhibition can render densely vascularized stromal regions more permissive to T cell migration. By depleting TAMs, these therapies reduce local cellular density and diminishes T cell retention, thereby facilitating intratumoral motility. Our initial study using dynamic live imaging provided direct evidence that CD8^+^ T cells exhibit increased motility following CSF1R inhibition, and that combining this treatment with PD-1 blockade further enhanced CD8^+^ T cell infiltration into tumor islets (12). Interestingly, a concomitant increase in CXCL9 was observed, suggesting that enhanced CD8^+^ T cell infiltration may result from both reduced retention by suppressive TAMs and increased chemoattraction mediated by CXCL9. As described above, CXCL9^+^ TAMs can already be found in tumors at variable densities. However, therapeutic interventions, including ICB (182), can markedly expand this population in sensitive tumors. The precise anatomical locations where these interactions occur during tumor rejection remain unclear but likely evolve as TILs progressively infiltrate tumor islets. Interestingly, in MSI CRC, exhibiting anti-tumor response, specific niches of TILs clustered with CXCL9^+^ TAMs and tumor cells have been observed at the tumor-stroma interface (59), and may represent a snapshot of an ongoing immune response. In line with this, during the early stages of tumor regression induced by a STING agonist in a murine breast cancer model, we found TAMs in close proximity to CD8^+^ T cells, which progressively exited the stroma and invaded tumor islets (186). IFN-γ produced by CD8^+^ TILs can establish a positive feedback loop that promotes CXCL9 production in TAMs and monocytes (186, 197). Furthermore, IFN-γ released by CD8^+^ TILs has been implicated in the upregulation of MHC-II expression in TAMs, as well as in the acquisition of cytotoxic functions against tumor cells (185). This interplay between TILs and TAMs creates a positive feedback loop that appears to be required, in certain settings, for the successful rejection of established solid tumors.

Crucially, functional studies have shown that this axis is essential for treatment efficacy in certain models: myeloid-specific deletion of *Cxcl9* completely abolished the anti-tumor effect of anti-PD-1 therapy (182), while blockade of its receptor, CXCR3, prevented T cell expansion within the tumor core (198). Together, these results indicate that the spatial coordination between TAMs and TILs may constitute a rate-limiting step for effective tumor regression.

Although considerable attention has focused on the role of TAMs in T cell recruitment, recent findings indicate that effector T cells actively shape supportive TAM niches. In models of successful immunotherapy, activated CD8^+^ T cells recruit monocytes and TAMs through the secretion of CCL5 (200). This proximity allows the macrophages to be polarized by T cell-derived IFN-γ, leading to iNOS expression and an immunostimulatory profile.

Whereas cytotoxic CD8^+^ T cells actively migrate through tumor islets to mediate direct tumor cell killing, other tumor regions may function as reactivation hubs for less differentiated anti-tumor T cells, sustaining the immune response over time. A recent study has shown that *in situ* TAM reprogramming can be pharmacologically enhanced using engineered cell microparticles. These microparticles, generated by extruding tumor cell membranes to retain tumor antigens and loaded with the dual TLR7/8 agonist resiquimod (R848), activate inflammatory signaling in TAMs, shifting them toward a pro-inflammatory state and promote their spatial proximity to TCF1^+^ stem-like CD8^+^ T cells, a critical population for durable ICB response (199). This engineered co-localization expanded the reservoir of stem-like cells and significantly increased the efficacy of anti-PD-1 therapy. This mechanism of attraction for activation implies that sustained anti-tumor immunity depends on T cells modulating TAMs to maintain the inflammatory milieu. Supporting evidence from *in vitro* co-cultures and human studies confirms this dynamic, revealing that TAM subsets linked to favorable ICB outcomes are precisely those participating in reciprocal activation loops with CD8^+^ T cells (200–202).

Targeting ECM components, remodeling enzymes, or matrix-sensing receptors on immune cells has emerged as a promising strategy to reprogram the TME and restore anti-tumor immunity. In the paper of Horn and colleagues, LAIR−1 and TGF−β blockade combined with anti−PD−L1 therapy reduced collagen-mediated barriers, promoting CD8^+^ T cell access and shifting macrophages toward a pro-inflammatory phenotype (203). Similarly, blockade of TGF-β signaling has shown potential to enhance T cell infiltration and improve responses to ICB (204). Different pharmacological and engineered ECM-remodeling strategies, including hyaluronidase-expressing oncolytic viruses and ECM-degrading CAR T cells, have shown to favor CD8^+^ T cell infiltration, reshape macrophage niches, and convert immunosuppressive microenvironments into immune-permissive ones, improving responses to ICB and other immunotherapies (205, 206). Future research should prioritize ECM-targeted therapies using spatially resolved, multiparametric analyses to map how ECM remodeling affects TIL-TAM niches *in vivo*. This understanding is essential to design next-generation combinations that restore effective anti-tumor immunity and achieve durable clinical responses.

## 5 Discussion and future perspectives

In this review, we propose that precision oncology should move from the simple quantification of individual cell densities, such as TIL infiltration, in favor of a multidimensional mapping of TAM-TIL niches. Shifting from a “cell-centric” to a “niche-centric” paradigm (**Figure 1**) allows us to integrate the spatial, phenotypic, and functional layers of these cellular crosstalk, providing the mechanistic rationale needed to understand why specific microenvironments resist or respond to immunotherapy and guiding personalized treatments. The next frontier in understanding the TME is the transition from descriptive spatial mapping to a multi-layered functional understanding of TAM-TIL niches and, more generally, of the interaction among cell populations. An important and exciting advancement in this field is the capacity to perform spatial multi-omics, specifically integrating transcriptomics, proteomics and metabolomics on the same tissue section. While spatial transcriptomics identifies the gene expression programs and cell states, such as the previously mentioned progenitor or exhausted populations, co-profiling with spatial metabolomics on a single slide reveals the actual biochemical activity within those same coordinates (207). This same-section integration is crucial for mapping how localized metabolic competition directly determines the transcriptomic reprogramming of infiltrating T cells and the polarization of nearby TAMs. This integration is made possible by new platforms that combine matrix-assisted laser desorption/ionization (MALDI-MSI) with high-plex transcriptomics (208). Recent research on hepatocellular carcinoma has used this dual approach to pinpoint a distinct interface zone at the tumor margin where strong immune-CAF interactions are correlated with upregulated lactic acid and polyunsaturated fatty acids. By driving lactic acid secretion through glycolysis, CAFs produce immunosuppressive environments that favor tumor invasion, establishing a clear functional connection between metabolic barriers and immune exclusion (207). Furthermore, lipid-associated macrophages in the tumor-adipose interface of breast cancer were discovered through spatial transcriptomics analysis of fatty acid and lipid distribution. LAMs display M2-like immunosuppression, increased phagocytosis, and activation of lipid metabolism, which drives immune exclusion and resistance to anti-PD-1 therapy (209). Comprehensive mapping of the TME also requires spatial epigenomics, as the success of immune cell reprogramming relies entirely on how well these cells can adapt to external stimuli. Using methods such as spatial Assay for Transposase-Accessible Chromatin using sequencing (ATAC-seq) to assess chromatin accessibility helps determine whether, for example, niche-specific TAMs have become epigenetically stuck in a suppressive phenotype or if they remain open to being therapeutically reprogrammed (210).

Beyond molecular data integration, volumetric considerations also need to be accounted for. Tumors are not flat entities and this reprogramming is also influenced by the 3D architecture of the tissue. Pentimalli et al, addressed this concern by reconstructing serial NSCLC sections into a 3D spatial multi-omic framework, demonstrating that tumors are not functionally uniform structures. This 3D shift revealed that different or even opposing niches can coexist in the same tumor, suggesting that metrics like traditional TIL count are not able to truly represent TME complexity (211). Consequently, together with cell density and niche quality, the 3D arrangement between immune populations and the architecture of the ECM is an important determinant of therapeutic potential.

The huge volume and high-dimensionality of integrated spatial multi-omic datasets require a shift toward AI-driven analytical frameworks able to go beyond simple cell counting to reveal the functional logic of the TME (212). Advanced platforms such as Novae, a foundation model based on graph neural networks and transformers, are revolutionizing how we handle these data. By using deep learning architectures trained on millions of cells, Novae enables the identification of immunity hubs directly from the data, without prior annotation or predefined interaction labels. Essentially, it can automatically detect complex spatial domains in new, unseen tissue samples without needing any prior, dataset-specific training (213). This enables rapid, unbiased mapping of active versus suppressive niches across diverse patient cohorts.

This computational scalability is complemented by clinically validated scoring systems, such as the Immunoscore-IC, which employs machine learning on digital pathology slides to quantify CD8^+^ T cell density, PD-L1^+^ cell density, and their spatial proximity across tumor core and invasive margin regions. While traditional pathology struggles with inter-observer variability, this automated deep learning approach segments immune and tumor cells via feature extraction, then computes interaction scores that reveal functional immune engagement (120). Such analysis has demonstrated superior prognostic value for predicting immunotherapy response in NSCLC compared to non-spatial pathologist assessments, enabling rapid TME stratification in heterogeneous clinical samples.

While TAM-TIL functional scores based on minimal genes/proteins panels (214) are an attractive translational target, their clinical implementation remains significantly limited by both biological and technical constraints. Biologically, the reliability of low-plex signatures is limited by profound intra-tumor spatial heterogeneity and the context-dependent roles of many markers; for this reason these signatures should be framed as spatially informed and validated across tumor regions and sampling methods (215). Unfortunately, integrating spatial parameters into routine clinical diagnostic is currently too time-consuming and cost-prohibitive for large-scale patient stratification. Moreover, current clinical instruments are not designed to perform spatial genomic profiling, restricting these analyses to research platforms requiring dedicated facilities and specialized personnel. For these reasons, translation efforts for the next future should focus on expanding the favorable neighborhood, either by delivering engineered myeloid and T cell populations, recruiting specific immune progenitors or using pharmacological agents to recruit, reposition or reprogram the existing TME components. At the same time, it is necessary to prioritize interventions that target the molecular drivers of suppressive niches to restore T cell function (216, 217). The final requirement is the development of multi-modal AI tools able to convert complex multi-omics into localized, actionable targets to strategically reprogram the immune neighborhood.

Looking forward, advanced computational modelling will be indispensable not as a replacement for biological interpretation, but as the only possible framework for the cross-modal integration of heterogeneous datasets. While the accuracy of these algorithms remains a critical area for ongoing refinement and rigorous clinical validation, the sheer scale of integrating same-section metabolomics, high-plex protein signatures, and the physical constraint of the ECM exceeds the capacity of traditional manual analysis. Advanced computational models are now being developed to combine complex spatial data with standard clinical parameters, allowing us to better predict patient outcomes and guide precision therapies. Ultimately, the synergy between same-section multi-omics and AI-assisted spatial analysis is what will allow us to pinpoint actionable “checkpoint niches”. By using these tools to precisely calculate the interaction markers, metabolic states, and physical accessibility within these hubs, we can start designing next-generation therapies, like bi-specific T cell engagers tailored for TAM-TIL bridges or localized metabolic modulators, specifically engineered to reshape the cellular dialogues at the tumor-immune interface. This shift from “cell-centric” to “niche-centric” drug development could truly redefine the scope of immunotherapy, moving the goal from activating a single cell type to reprogramming the entire cellular neighborhood.
